# New Orleans in May

**Published:** 1858-02

**Authors:** 


### HALL'S JOURNAL OF HEALTH.

OUR LEGITIMATE SCOPE IS ALMOST BOUNDLESS: FOR WHATEVER BEGETS PLEASURABLE AND HARMLESS FEELINGS, PROMOTES HEALTH; AND WHATEVER INDUCES DISAGREEABLE SENSATIONS, ENGENDERS DISEASE.

WE aim to show how disease may be avoided, and that it is best, when sickness comes, to take no medicine without consulting an educated physician.

VOL. VI.] FEBRUARY, 1858. [No. 2.

### NEW ORLEANS IN MAY.

HAVING spent a decade of our life in the Crescent City, we may be considered as speaking "by authority," the authority of personal experience and observation, when we pronounce the opinion, that the month, including the first day of May, is the safest, healthiest, and most delightful season in the whole year, to all strangers from a more Northern latitude. Safe, because the Mississippi river is always high at that time, and the low lands are so deep under water, that the great agent of disease and death, *Miasm*, is not generated. It is in the latter part of Summer and earlier Fall, when low waters expose to a hot sun thousands of square miles of shallow marsh bottoms, that pestilence and deadly fevers march multitudes to the grave. Heavy damp weather is, in all latitudes, the most disagreeable, and is the fruitful cause of depressing, although not fatal maladies; but about the first of May, the atmosphere is balmy and delightful, and what showers there are, soon dry up; the chilly Spring has entirely passed away, and no fires are by any possibility needed.

At the same time, persons arriving from the North should bring their light umbrellas, which will answer the double purpose of *paraplui* and *parasol*, protections against shower and sun; they should also bring their thin woollen hose, and wear next the skin the same flannels they had on leaving home—for two reasons: to protect them from the evening chilliness, and as safeguards against cooling off too soon after walking; for strangers are very apt to walk so fast as to excite considerable perspiration, before they know it, and the breeze which springs up oceanward, towards evening, cools off very rapidly, to say nothing of the strong temptation, when warm, to pull off the hat, and sit on the universal piazza, or at an open door or window.

The second precaution worthy of mention is, eat what you please, and as much as you want for breakfast and dinner, which last is commonly about three o'clock; but make it an imperative rule, not to eat an atom of any thing between breakfast and dinner, nor after dinner either, except an orange or two.

Another precaution: Do not, on any account, go outside of the door of the house, in which you have slept, without either eating your regular breakfast, or taking at least a cup of hot coffee, which, if you cannot conveniently get in the house, you will find ready prepared at some of the street corners.

It was both a wise and beneficent dispensation, that man should have been made capable of eating anything, and of living anywhere, and living, too, in comparative enjoyment. *Livingston*, the benefactor of commerce, and *Howard*, the benefactor of man, have shown by their lives, that health may be maintained in any country, by those who were not born to the soil, at the expense only of rational care.

To insure safety then, against any attack of sickness, in New Orleans, in the Spring of the year, visitors

1. Must locomote in stately slowness.
2. Must deny themselves all lunches, and all eating between meals.
3. Must eat nothing after a three o'clock dinner, but a few oranges.
4. Must avoid going out of doors early in the morning, unless something is eaten, or some warm drink taken.

By a rational attention to these four things, a Northerner will be as safe in New Orleans, from November until July, as he would be at his own home, under ordinary circumstances. It is not the climate of New Orleans which is so destructive of human life. It is the three Ls: the Liquor, the License, and the Late supper, which make their annual hecatombs. We know, personally, many Northerners, who have gone there, and in the course of years have accumulated fortunes, and left in excellent health, or remain to enjoy both health and fortune; but there is not a wine drinker, nor a gourmand, nor a *latitudinarian* among them, not one; every one of them is a man of steady habits, who had his Northern principles of systematic moderation in all things, and maintained them. Personally we never had better health, than when a resident there, even at a time when the cholera was numbering its daily hundred victims, dying all around our habitation and our office. And why?—we took no liquor *and no medicine!* kept regular hours, ate what we wanted, as much as we wanted, *and no more.*

It may be useful to give the reason for the precautions named, as a means of impressing the memory as to their necessity.

Why you should walk slowly in warm weather, needs no explanation to thinking people; but, as nine-tenths of mankind never think, but act mechanically as to the commonest facts of life, we may state, that walking fast in Summer time causes perspiration; and if, while in that condition, a person is stopped in the street, or in any way exposed to a draft of air, a cold is inevitable.

We should not eat between meals, in warm weather especially, because we all feel weaker than in cold weather, and the stomach has its share of that debility, and it is no more capable of working all the time without rest, than a man in midsummer could work incessantly without rest, even during the hours of daylight. But, when we take an ordinary meal, it requires at least four hours' labor for the stomach to digest it, and send it out to another part of the body; or if we eat an apple or a cracker or sweet cake, it takes one, or two, or three hours for the stomach to digest it. It is easy to see then, that if a man takes a regular breakfast at eight o'clock, and then a lunch at twelve, and a dinner at three, or four, or five, the stomach is kept in incessant operation from eight in the morning until the close of the day; and the stomach is a muscle, or rather a collection of muscles, and it can no more be kept in exercise all day with impunity, than the hand or foot, or any other movable part of the body. Continuous work or walking will kill any man, will debilitate him beyond resuscitation—and that is *Dyspepsia*, as applied to the stomach; it has been worked so hard, so much, so incessantly, that, like an overworked or overtravelled animal, all the beating, all the goading in the world, will not rouse it up. Now, if any man after this, fails to comprehend in its fullest sense, what the famed word *Dyspepsia* is, he is hopelessly daft, and he had better not take this Journal any longer, for we can't teach him.

It is important to remember, that Dyspepsia is of two kinds; one called *chronic*, which lasts for a month, or a year, or a life-time; this is the kind to which the familiar word "Dyspepsia" applies; but, there is another kind, *acute*, which may be brought on in any twenty-four hours, and run its course to a fatal termination within the same time, in the shape of cholera morbus, cramp colic, bilious colic, and child epilepsy, convulsions, and "fits"—these being the results of *Acute Dyspeptia*, of giving the stomach, at one meal, more than it can possibly do, or of repeating its task for a whole day, before one is completed; and to do this in hot weather, when the stomach and all the body are at their weakest, is nothing less than suicidal: and this is the chief reason why, even in New York, there are nearly double the number of deaths in midsummer that there are in midwinter—the stomach is worked to death, by the three regular meals and eating between times.

We place great stress on taking some food or stimulating drink into the stomach, on waking in the morning, before going out into the morning air, in all Southern latitudes, especially in the warm weather, because we all wake up in a languid condition; the stomach naturally, and by means of its long fast, has its share of languor, and has almost no power of resisting its own instinct to drink in whatever is presented to it; nor have the other parts of the system any greater ability of self-defence, of resisting deleterious impressions from an atmosphere loaded with poisonous miasm, which is present in its greatest malignity and in its most concentrated and compact form for the hour or two about sunrise, in warm weather, in all Southern latitudes—malignant enough, in some localities, to cause death in forty-eight hours. A little food, or a cup of hot drink, wakes up the weak stomach, imparts nutriment to it, and with that, strength to defend itself. Hence, all persons should take their breakfast before they travel in warm weather; and, for the same reason, all out-door laborers, farmers, &c., should do the same thing. It is to the habit of taking a cup of coffee, even before getting out of bed in the morning, in many instances, that the "Creoles," that is, the native-born of Louisiana, owe their impunity against Southern diseases, so much above others of the population, whom ignorance, inattention, prejudice, or foolhardiness prevent from observing the time-honored custom.

What we have thus said about a safe manner of spending a May in New Orleans, is applicable in all lands between the temperate zones, in warm weather, and attention to them would save millions of lives every year.

